# Evolutionary Patterns of Intersexual Power

**DOI:** 10.3390/ani13233695

**Published:** 2023-11-29

**Authors:** Rebecca J. Lewis, E. Christopher Kirk, Ashley D. Gosselin-Ildari

**Affiliations:** 1 Department of Anthropology, University of Texas at Austin, 2201 Speedway, Stop C3200, Austin, TX 78712, USA; eckirk@austin.utexas.edu; 2 Independent Researcher, Durham, NC 27705, USA; agosselinildari@gmail.com

**Keywords:** male dominance, female dominance, codominance, sexual dimorphism, leverage, inequality, social evolution, fossil, primate evolution

## Abstract

**Simple Summary:**

Social inequality is pervasive in primates, with male-biased power often assumed as the default condition. We tested this assumption with a broad dataset and explored factors that might make some types of intersexual power more likely to evolve, such as males being larger than females and skewed sex ratios. We hypothesized that intersexual power can arise due to sex differences in fighting ability as well as economic demand for mating opportunities. We found that even though societies with power biased towards males are most common in primates, some lemurs, monkeys, and apes exhibit societies without male-biased power. Male-biased power was associated with males being larger than females and more females in social groups (i.e., high supply/low demand for females), whereas non-male-biased power was associated with low supply/high demand for mating opportunities with females. The last common ancestor of primates did not necessarily exhibit male-biased power, but the last common ancestor of monkeys, apes, and humans probably did exhibit male-biased power. Non-male-biased power probably evolved independently multiple times in primates. However, multiple traits favor male-biased power and fewer paths lead to societies with female-biased power or intersexual equality.

**Abstract:**

Dominance and leverage are both possible causes of social inequality. If sexual dimorphism influences patterns of intersexual dominance, we predicted that highly dimorphic species are constrained to exhibit male-biased power (MP), but species with low sexual dimorphism are free to demonstrate a broader range of intersexual power relationships. If market effects influence intersexual leverage, we predicted that females have more power when group composition is more male-biased and estrus is asynchronous. We analyzed data on intersexual power, sexual dimorphism, expected estrous overlap, and sex ratio for 79 extant primate species using phylogenetic logistic regression and ancestral state reconstructions. Although MP is more common, every major primate clade includes non-MP species. MP was associated with greater body mass and canine length dimorphism and with female-biased sex-ratios. Low estrous overlap was associated with non-MP. Although MP was reconstructed as likely ancestral for anthropoids, the last common ancestor of this clade probably did not exhibit high sexual dimorphism. The last common ancestor of catarrhines was probably highly dimorphic, potentially constraining intersexual power relationships. Non-MP probably evolved multiple times in primates and may be less common because multiple traits are linked to MP while fewer traits are associated with female-biased power or equality.

## 1. Introduction

Power occurs when an inequality exists in a relationship [1,2]. It can arise from many different types of asymmetries, including fighting abilities, coalition partners, inalienable resources, dependency, and knowledge [2,3,4]. When power arises due to an asymmetry in the ability to use physical force, it is termed “dominance”, but when power arises due to an asymmetry in economic power, it is termed “leverage” [2].

Intersexual power may be broadly defined as the ability of an individual to influence the behavior and/or physiology of another individual of the opposite sex based upon an asymmetry in that dyadic relationship [3,5]. In mammals and birds, intersexual power has been theorized to have a reciprocal relationship with sexual dimorphism, in which sexual dimorphism influences, and in some cases may be influenced by, species-typical patterns of intersexual power [6,7,8,9,10]. Specific patterns of intersexual power have also been attributed to sex ratio [3,9], seasonality [9,11], energetic costs [12], selection for deferential mates [10,11,13,14], intersexual differences in social support [15,16], winner–loser effects [17], and frequent intersexual interactions [18]. Intersexual power can also arise due to female control over reproduction [19,20] and the supply and demand (i.e., market effects [21]) of reproductive opportunities [2,3].

Primates [22] and other mammals [8,23] are reported in the literature to mostly exhibit male-biased power structures, in which males have a greater ability than females to influence the behavior and physiology of their opposite-sex social partners. (Our use of the terms “male-biased power structure” and “female-biased power structure” refers specifically to the social structure (*sensu* [24]) observed in a species rather than a dyad, consistent with the power framework [2,3,4]. These terms are preferable to “female dominance” and “male dominance” because they allow for the broad range of possible power asymmetries [5].) Often overlooked, however, is that research on mammals is biased towards larger taxa [8,25], and sex-biased power structures are not present in all mammals [8], such as when taxa are not social or because they exhibit co-dominance (i.e., the power structure is not clearly biased towards one sex (for review of definitions: [5])). The prevalence of female-biased power structures in the lemuriform primates is unusual [26] and has sparked extensive debate (for review: [5]), with some authors suggesting that female-biased power is a trait that evolved in their last common ancestor [26,27]. However, power is not a characteristic of an individual but rather a characteristic of a relationship [2,4,28,29] because the same individual can be simultaneously dominant and subordinate depending on the social context. Selection can nevertheless act on the traits of individuals that influence social relationships [2,4]. Thus, if female-biased power structures are a synapomorphy (a derived trait that is shared by multiple taxa; see Section 2.3 for definitions of cladistic terminology) of some primate clades, then they must have evolved as the result of selection for the trait(s) of individuals that create a critical asymmetry in intersexual dyads [3].

Multiple authors have discussed what is now termed the “lemur syndrome” of female-biased power structures, monomorphism, and even adult sex ratios [9,30,31] and attributed this suite of characteristics to the unique ecology of Madagascar (e.g., [12]). Nevertheless, female-biased power structures occur in mammals not inhabiting Madagascar (e.g., spotted hyenas: [15,16]; review: [32]) and are the norm in some vertebrate taxa (e.g., raptors [10]). Consequently, a broader explanation for the evolution of female-biased power structures is required.

We examined the evolutionary relationship of sexual dimorphism, market effects, and interspecific patterns of intersexual power in primates because primates exhibit a great diversity of social and mating systems, body sizes, and power relationships [5,33]. Note that our use of the term “sexual dimorphism” refers specifically to male-biased sexual dimorphism, in which males are larger than females. Although some primate species exhibit low degrees of female-biased sexual dimorphism (in which females are larger than males), high degrees of female-biased sexual dimorphism do not occur in primates and are uncommon in mammals generally [8,25]. Additionally, a densely sampled primate fossil record facilitates the reconstruction of patterns of sexual dimorphism over time. We categorized 79 primate species as having either (a) male-biased power structures or (b) power structures that are not biased towards males based on whether the published literature labeled the species as “male dominant”, “co-dominant”, or “female dominant” (online Supplementary Materials Table S1). Although we here utilize the power framework [2,3,4,5], which specifically defines “dominance” as a phenomenon arising from an asymmetry in the ability to use force in a dyad, this framework was not used by the publications in our dataset. Therefore, our use of the terms “male dominance”, “co-dominance”, and “female dominance” here refers to the authors’ labels and not necessarily the definition of dominance in the power framework. We conducted Ancestral State Reconstructions (ASR) for discrete power categories and sexual dimorphism, and then used the predicted probabilities from phylogenetic logistic regressions of sexual dimorphism to reconstruct likely ancestral patterns of power.

We hypothesized that both dominance and leverage (*sensu* [2,3,4]) influence sex-dependent power in primates. If sexual dimorphism does exert a causal influence on species-typical patterns of intersexual power, then we predicted that highly dimorphic species are constrained to exhibit mainly male-biased power structures because larger body size might favor male dominance (i.e., power based on force or the threat of force) in intersexual dyadic relationships. We further predicted that species characterized by low degrees of dimorphism are not similarly predisposed to male dominance and are therefore free to demonstrate a broader range of intersexual power structures influenced by variables other than sexual dimorphism. If intersexual power is based on leverage, then we predicted that power varies with sex ratio and estrous synchrony due to market effects [2,19,21,34,35]. More specifically, we predicted that females have more power when a social group is comprised of more males relative to females because the less common sex should have more power according to the expectations of supply and demand for mating partners. Additionally, if the supply and demand of estrous females is a source of female power, then female power is expected to be associated with estrous asynchrony, as measured by expected estrous overlap [36,37]. In other words, a female who is the only individual in estrus in her social group is expected to have greater power in her dyadic interactions with males compared with a female who is one of multiple estrous individuals in her social group. Many extrinsic factors can influence sexual dimorphism, particular sex ratios, and estrous overlap [33,37,38,39]. Because we were interested in the consequences of these factors rather than their causes, the causes are not examined here.

## 2. Materials and Methods

### 2.1. Data Collection

The intersexual power structure of 79 primate species was collected from the literature with species initially designated as male dominant, female dominant, or co-dominant (Table S1). We scored species based upon the authors’ own assessment, even though this resulted in a dataset with inherent limitations, for several reasons. Importantly, the definition of dominance is not standardized in ethology [2,29,40,41]. Definitions of sex-dependent power are similarly inconsistent (for review: [5]). By limiting our study to primates, we were able to reduce some of the variation resulting from order-specific terminology (e.g., male “control” of female “harems” in ungulates [42]). The definition of female dominance is hotly debated and includes female feeding priority, male submissiveness towards females, and female aggressiveness towards males [9,22,27,43,44]. Co-dominance is often undefined but has been applied when females form coalitions against males and when no clear sex-dependent power exists [18,45,46,47]. Finally, male dominance is typically a baseline assumption in mammals, and consequently, poorly characterized [5,9]. While precise definitions are preferable in comparative analysis, species labeled as “female dominant” and “co-dominant” exhibit very different intersexual social relationships than species labeled as “male dominant”. For most analyses, we compared male dominant versus non-male dominant taxa, thereby reducing the importance of the exact definition of female dominance and co-dominance and instead focusing on whether taxa with these labels resemble taxa labeled as male dominant. Furthermore, we termed these two categories as ‘male-biased power’ and ‘non male-biased power’ in order to be more inclusive and account for the possibility of leverage.

Variables analyzed in relation to power (henceforth “power variables”) included body mass ratio (BMR), canine ratio (CR), expected estrous overlap (EO), and sex ratio (SR) and were collected from published literature (Table S1). BMR is defined as the average male body mass divided by the average female body mass. Body mass data were collected from [48], supplemented with data from [49]. CR is defined as the average male upper canine crown height divided by the average female upper canine crown height. Canine measurements were collected from [50], supplemented with data from [51,52]. EO is defined as the probability that two females in the same social group were simultaneously in estrus and was calculated following [37]. EO data were collected from [36]. Sex ratio is defined as the number of adult males in a foraging group divided by the number of adult females in a foraging group, and was collected from [36].

### 2.2. Statistical Analysis

To test the hypothesis that a given power variable is associated with a particular type of intersexual power, we ran a series of phylogenetic logistic regressions. Logistic regression is the appropriate statistical model for testing the association between continuous predictor variables (such as body size dimorphism or sex ratio) and a discrete dependent variable (intersexual power). Ref. [53] developed a logistic regression analysis that includes a phylogenetic variance–covariance matrix to account for species autocorrelation. This analysis estimates (1) the strength of the phylogenetic autocorrelation (i.e., “phylogenetic signal”) using an evolutionary model in which the binary (dependent) variable switches between states 0 and 1 as species evolve up the phylogeny and (2) the association between the continuous predictor variable and the probability that any species will be in state 0 or 1 [53].

Phylogenetic variance–covariance matrices were created using PDAP version 6.0 [54] and pruned versions of the primate phylogeny proposed by [55]. Phylogenetic logistic regressions were run using the Plog.Reg.m function [53] in MatLab version 8.4 (R2014b). Prior to analyses, all variables were log10 transformed. As recommended by [53], continuous predictor variables were standardized such that the mean equaled 0 and the standard deviation equaled 1. This standardization results in regression coefficients that are representative of effect size [53]. The authors in [53] note a bias in regression coefficients when phylogenetic signal in the residual variation is high and recommend parametric bootstrapping for parameter estimation. We performed parametric bootstrapping to estimate parameters, significance levels, and confidence intervals. Bootstrapping was performed for 2000 simulations, and the alpha-level was set at 0.05. Convergence of the bootstrapping was achieved in all analyses.

We used the PLogReg.m function to test for an association between the power variables and the odds of a species exhibiting male-biased power. Male-dominant species were coded as 0, and female- or co-dominant species were coded as 1.

To determine if high levels of body size dimorphism exhibited by catarrhines constrains the relationship between power variables and intersexual dominance, additional logistic regressions were run for EO and SR. In these analyses, all taxa with BMR greater than 36% were removed (Table S1).

### 2.3. Ancestral State Reconstruction

Ancestral state reconstruction (ASR) analysis is commonly used across comparative biology and paleontology to test evolutionary hypotheses about changes in discrete or continuous morphometric and behavioral variables across a given clade. ASR analysis can use a variety of statistical models, including generalized least squares, maximum likelihood, and Bayesian inference, to estimate values for internal nodes on a phylogeny using the measured values for extant (and sometimes fossil) taxa at the tips of the tree [56]. In addition to reconstructing ancestral values, ASR analyses are used to study the evolutionary changes in a trait over time. 

Describing evolutionary changes across lineages requires precise terminology. These terms may refer to groups of species, including *clade* (a group that contains the last common ancestor [LCA] of a group of species and all of the descendants of that common ancestor), *crown* taxon (a member of a clade defined by living taxa), and *stem* taxon (an extinct taxon that is outside the crown group but is more closely related to that crown group than any other clade defined by living taxa). Additional terms refer to the characteristics of taxa, including *primitive* or *plesiomorphic* traits (older traits inherited from a more distant taxon than the LCA of a clade), *derived* or *apomorphic* traits (novel traits that were newly-evolved in the LCA of a clade), and *synapomorphy* (a derived trait that is shared by multiple taxa).

All analyses were run in R Statistical Software (v3.1.0; [57]). We ran one ASR analysis using our discrete power categories and several ASR analyses using our continuous variables expected to be associated with intersexual power (BMR, CR, and SR). All ASR analyses used a maximum likelihood estimation (MLE) model to reconstruct ancestral states at internal nodes along a phylogeny from the data of terminal taxa. We present reconstructed ancestral estimates for the last common ancestors (LCAs) of crown Primates, Strepsirrhini, Haplorhini, Lemuriformes, Lorisiformes, Anthropoidea, Platyrrhini, Catarrhini, Cercopithecoidea, and Hominoidea in Table 1. 

The ASR analysis for discrete power categories was run using the ace function in the *ape* package (v3.1-2; [58]), which follows the method outlined by [59] for studying phylogenetic correlation between discrete characters. For this analysis, taxa were coded as male dominant, co-dominant, or female dominant according to the data collected and presented in Table S1. The phylogeny used in this analysis is the same as the phylogeny used in our logistic regression analyses. The output from the ASR analysis is nodal reconstructions that are scaled so that the total likelihood of the studied trait is equal to one (*ape* package, v3.1-2). The scaled likelihoods for male dominance, co-dominance, and female dominance at the ten LCAs discussed above are presented in Table 1. 

The ASR analyses for our continuous power variables were run using the *geiger* (v2.0.3; [60]) and *phytools* (v0.4-56; [61]) packages. We used the fast.Anc function in *phytools* to calculate a maximum likelihood estimate (MLE) and 95% confidence intervals [61] for the ten LCAs using the continuous data from our power variables and corresponding phylogenies. A separate ASR analysis was run for each power variable. These three analyses used the same datasets and pruned phylogenies used in the phylogenetic logistic regressions. LCA nodal reconstructions for BMR, CR, and SR are presented in Table 1.

Subsequently, we ran an additional ASR analysis for both our BMR and CR power variables. Eight euarchontan outgroup taxa (Table S2) from Dermoptera and Scandentia were added to the BMR dataset, and an ASR analysis was run to determine the changes, if any, to the MLE of our internal nodes, especially the LCA of Primates. The genera *Galeopterus* and *Cynocephalus* have previously been reported as monomorphic, and we assigned a BMR of 1.0 to these taxa [62]. Body masses for male and female scandentians were collected from [63,64]. Although body mass data are available for these taxa, the intersexual dominance status of these species is unknown and therefore precluded from inclusion in the phylogenetic logistic regression. These eight taxa were added to the pruned [55] phylogeny. Branch lengths for Euarchonta, Primatomorpha, and Dermoptera crown node divergences were based on [65] and obtained from TimeTree [66]. Branch lengths for crown Scandentia and all other Scandentian nodes were based on [67] and obtained from TimeTree [66].

Eight fossil euprimate taxa with reported canine ratios were added to our CR dataset, and an ASR analysis was run to determine the changes, if any, to the MLE of our internal nodes, especially the LCA of Primates, Strepsirrhini, Anthropoidea, Haplorhini, Platyrrhini, and Catarrhini. These fossil taxa include the stem strepsirrhines *Leptadapis magnus*, *Adapis parisiensis*, *Northarctus venticolus*, and *Cantius torresi*, the extinct haplorhine *Teilhardina belgica*, the stem anthropoid *Proteopithecus sylviae*, the stem catarrhine *Catopithecus browni*, and the stem platyrrhine *Homunculus patagonicus*. Although *Teilhardina* is likely either a stem tarsiiform or a stem haplorhine, its placement as a stem anthropoid (basal to *Proteopithecus*) in our analyses was necessitated by the absence of tarsiers in our CR dataset. Tarsiers were not included in these analyses because their canine ratios have not been reported previously. Although qualitative descriptions of canine sexual dimorphism have been reported for more than these eight fossil euprimates [68,69,70,71,72,73,74], we chose to mainly include fossils where measurements of canine crown height were available. The only fossil taxon included that did not have reported canine lengths was the omomyoform *Teilhardina belgica*. Omomyiforms have previously been reported to be monomorphic in canine ratio [75,76], and we chose to include *T. belgica* as a representative omomyiform for two reasons. First, canine dimorphism is common throughout the anthropoid clade, and a potential stem lineage exhibiting monomorphism may affect the results of ASR analyses. Second, our analyses included up to four adapiforms as stem strepsirrhines and we considered an early representative of the haplorhine lineage an important inclusion.

The methods used to place fossil taxa in our tree (Figure S1) and set branch lengths follow [77,78]. Specifically, we computed the divergence date (Table S3) between each fossil taxon and its sister taxon or clade using data on genetic divergences within crown primates, the estimated geological age of the fossil, and the hypothesized relationships of the fossil to extant lineages. Ages of fossil taxa were based on absolute and relative dates of the localities from which the fossil taxa are known (Table S3). When fossil taxa were substantially younger than their sister taxon/clade, they were assigned a branch length equal to the difference between their age and that of their sister taxon, plus a one-million-year buffer. This situation applied to *Northarctus venticolus*, *Cantius torresi*, *Leptadapis magnus*, *Adapis parisiensis* (stem strepsirrhines), *Proteopithecus sylviae* (stem anthropoid), and *Homunculus patagonicus* (stem platyrrhine). The divergence between *Leptadapis* and *Adapis* may be as old as ~43 million years ago based on material known from the Egerkingen locality in Switzerland [79]. The known age for *Catopithecus browni* (stem catarrhine) precedes the divergence of crown catarrhines in the genetic phylogeny, and it was given a one-million-year branch length. Though *Teilhardina belgica*’s age also precedes genetic divergence of its presumed crown sister taxon (anthropoids), it was not given a one-million-year branch length. Instead, *Teilhardina belgica* was used to reflect the genetic divergences of *Tarsius* from anthropoids. Tarsiers themselves could not be included in CR analyses because we lack data on maxillary canine crown height dimorphism in tarsiers.

We chose not run additional BMR ASR analyses using reconstructed body masses of fossil euprimates. While canine length can be directly measured from fossil jaws, the body mass of fossil taxa is an estimate based on predictive equations that are often accompanied by large error ranges [80,81].

Six different models of evolution were tested for each ASR analysis using continuous power variables outlined above. We used the fitContinuous function in the *geiger* package [82] for model fitting. The models included Brownian Motion, lambda, kappa, delta, Ornstein-Uhlenbeck, and early-burst. Models that did not achieve convergence were excluded. Log–likelihood ratio tests were used to determine if the complex models were a significantly better fit to the data than the Brownian Motion model. Complex models that had a significantly better fit than the Brownian Motion model were compared using the Akaike Information Criterion (AIC) [56]. The parameters of the models with the highest AIC values were used to apply a branch length transformation to the phylogeny prior to ASR analysis using the rescale function in the *geiger* package [82]. Ancestral state reconstructions were run with the fastAnc function in the *phytools* package [61].

### 2.4. Predicting Probability of Exhibiting Male-Biased Power along Phylogeny

A series of predictive equations to calculate odds of being male dominant at the ten LCAs were generated using the output from our logistic regression analyses. Odds were calculated as *exp*(*b*_0_ + (*node MLE* × *b*_1_)) where b_0_ is the bootstrapped mean of the intercept, *node MLE* is the maximum likelihood estimate for an internal node, and *b_1_* is the bootstrapped mean of the independent variable. Values for *b*_0_ and *b*_1_ used in our predicted equations are listed in Table 2. Using the odds calculated from our predictive equations, a predicted probability of being male dominant was calculated using the equation *odds*/1 *+ odds*. Predicted probabilities of being male dominant at the ten LCAs are presented in Table 1.

One limitation should be noted about the ASR analyses that include outgroup and fossil taxa. The logistic regressions presented in Table 2 are based on a dataset and phylogeny including only extant primates. Predictive equations generated from these logistic regressions were used to predict the likelihood of male dominance from ancestral nodes estimated using ancestral state reconstructions. However, some of these ancestral state reconstruction analyses included data on outgroup taxa and fossil primates (specifically, BMR and CR). The results from these analyses were generated using predictive equations that are based on logistic regressions that do not include the outgroup or fossil taxa. Furthermore, we note that Table 1 presents the MLEs of reconstructed nodes and the predicted probability of male power from the ASR analyses of BMR, CR, SR using the original extant primate dataset because no substantial differences were found between these three ASR analyses and the ASR analyses that included the extant outgroup genera Dermoptera and Scandentia (BMR) or the fossil primate taxa (CR).

## 3. Results

**Figure 1:**
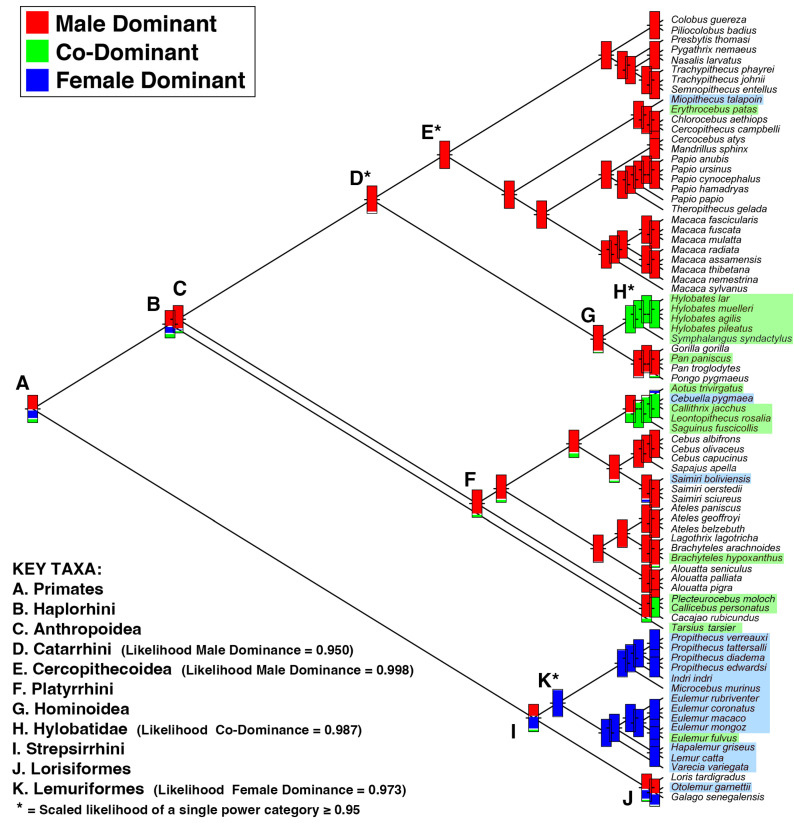
Intersexual power is highly variable in primates. Colored boxes indicate type of intersexual power reconstructed for ancestral nodes in the ASR analysis of discrete power categories. In each box, the width of the color band (red = male dominant, green = co-dominant, blue = female dominant) is proportional to the scaled likelihood of each power category occurring at the node. The character state of a clade’s last common ancestor (LCA) is unambiguous when the box is a solid color. Extant taxa with non-male-biased power are highlighted on the right (green = co-dominant, blue = female dominant). Key nodes are identified by capital letters. * identifies nodes with a scaled likelihood of ≥0.95 of a single power category occurring at the node. Male-biased power is more likely in the LCA of Anthropoidea, while female-biased power is more likely in the LCA of Lemuriformes. Greater uncertainty exists for the LCA of Primates, Strepsirrhini, and Haplorhini. If the LCA of Anthropoidea exhibited male-biased power (scaled likelihood = 0.844), then the various anthropoid taxa that do not exhibit male-biased power are probably derived and document >7 transitions to power that is not biased towards males.

The majority of the primate species in our sample (i.e., 58%) exhibit male-biased power structures. However, the number of species described as having male-biased power structures varies considerably by clade, with male-biased power most common among catarrhines and entirely absent among extant lemuriforms. Furthermore, even within Catarrhini, both Cercopithecoidea (e.g., *Erythrocebus*, *Miopithecus*) and Hominoidea (hylobatids, *Pan paniscus*) include multiple taxa that do not exhibit male-biased power (Figure 1). Among the larger clades in our sample, Platyrrhini exhibits the greatest variability in intersexual power structures, with approximately 40% of species categorized as co-dominant or female dominant. In our ancestral state reconstructions, the most likely intersexual power structures of the largest clades (i.e., Primates, Haplorhini, and Strepsirrhini) are currently unresolved (Table 1). Greater certainty about the ancestral pattern exists for some clades: the last common ancestor (LCA) of Lemuriformes likely exhibited female-biased power, while the LCA of Anthropoidea, Platyrrhini, and Catarrhini likely exhibited male-biased power. These ASR results suggest that the anthropoid taxa not exhibiting male-biased power are probably derived in this respect and that a transition from male-biased power to co-dominance or female-biased power occurred at least seven times within the Anthropoidea (Figure 1). Consequently, explanations for the evolution of primate intersexual power need to account for the parallel acquisition of non-male-biased power in multiple anthropoid clades.

**Figure 2:**
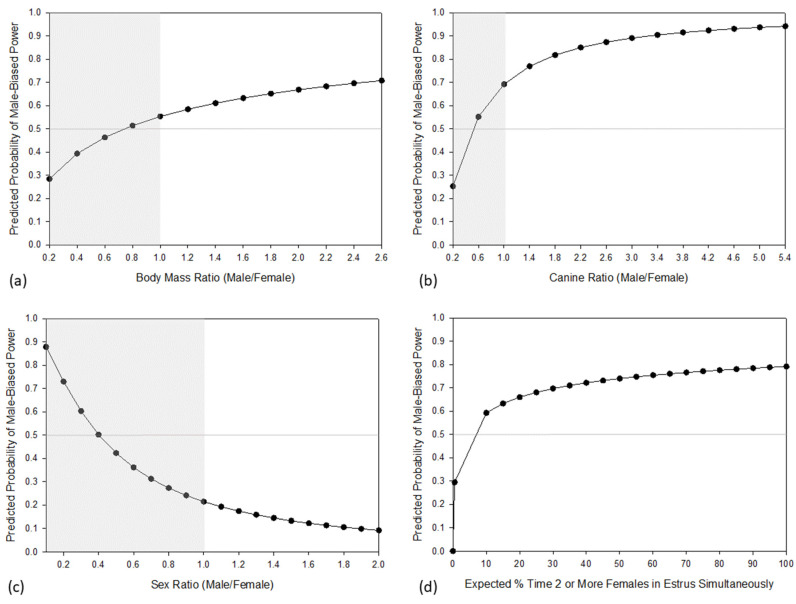

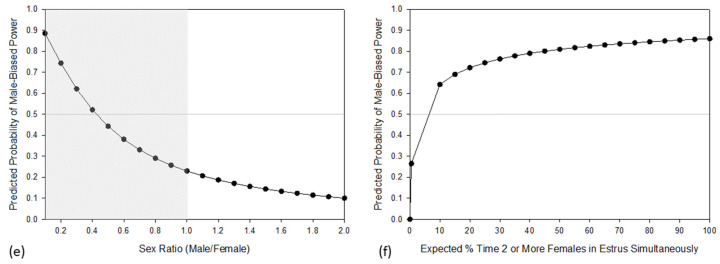
Predicted probabilities of male-biased power. The predicted probability of male-biased power increases with increasing dimorphism in (**a**) BMR and (**b**) CR. Similarly, the predicted probability of male power is ≥50% when (**c**) the sex ratio is greater than about 0.4 and (**d**) estrus is almost entirely asynchronous. The potential effect of female leverage increases only very slightly when extremely dimorphic species are excluded (**e**,**f**). Note that these predicted probability plots include extrapolations outside the range of *x*-axis values observed in primates. In our dataset (Table S1), BMRs of extant primates range from 0.85 in *Indri indri* to 2.45 in *Mandrillus sphinx,* and CRs of extant primates range from 0.88 in *Propithecus edwardsi* to 5.18 in *Mandrillus sphinx*. The horizontal gray line indicates the 50% probability threshold on the *y*-axis. Grey boxes highlight female-biased dimorphism (**a**,**b**) or female-biased sex ratios (**c**,**e**).

We next examined whether fighting ability and/or supply and demand of mating opportunities predicts patterns of intersexual power in primates. Our results support the initial expectation that fighting ability influences intersexual power (Table 2). Male-biased power was significantly associated with greater sexual dimorphism in body mass and canine length. When males are substantially larger than females, primate societies tend to be male dominant. As sexual dimorphism decreases, the predicted probability of a species exhibiting male-biased power also decreases (Figure 2a,b). Nevertheless, the predicted probability of exhibiting male-biased power was greater than 50% even when males and females are monomorphic, although at approximately 55%, the predicted probability of male-biased power is close to parity at monomorphism. Inclusion of non-primate euarchontan outgroups and of fossil primate taxa had a negligible influence on our analyses of BMR and CR (respectively). Dimorphism in both body mass and canine length was reconstructed as relatively low in the LCA of Primates, with confidence intervals of MLEs including 1.0 (monomorphism) at this node for both BMR and CR (Table 1). If this reconstruction is correct, then intersexual power in the primate LCA would not have been constrained by sexual dimorphism, and male-biased power should therefore not be assumed as a baseline expectation for the primate LCA. Much higher dimorphism in body mass and maxillary canine size was reconstructed for the LCA of Catarrhini (Table 1), suggesting that this clade was more likely to have been constrained to exhibit male-biased power. Interestingly, within catarrhines, four taxa diverge from the ancestral pattern of male-biased power: *Miopithecus*, *Erythrocebus*, hylobatids, and *Pan paniscus*. Of these taxa, extant hylobatids uniformly exhibit BMRs that are substantially lower than the MLE of BMR reconstructed for the LCA of Hominoidea. These data suggest that reduced sexual dimorphism evolved in the hylobatid stem lineage in concert with the evolution of non-male-biased power structures. Conversely, *Erythrocebus* has a substantially greater BMR than the MLE of BMR reconstructed for the LCA of Cercopithecoidea. This result suggests that a non-male-biased power structure in *Erythrocebus* unexpectedly evolved in concert with evolutionary increases in sexual dimorphism.

Our results further suggest that market effects may also influence intersexual power in primates (Table 2). Greater expected estrous overlap and female-biased sex ratios, both of which are expected to be associated with less female power in the mating market because the supply of mating opportunities for males is high (cf. [21]), were significantly associated with male-biased power. Estrous asynchrony and more equal sex ratios were associated with power that is not biased towards males. As sex ratio becomes more female-biased, the probability of male-biased power increases, but the shift to a greater than 50% predicted probability of male-biased power does not occur until the number of males is more than double the number of females (Figure 2c). In species with equal sex ratios, the predicted probability of male-biased power is quite low (~20%). Although the predicted probability of male-biased power changes with expected estrous overlap according to our initial expectations (Figure 2d), the effect of expected estrous overlap is more modest than for sex ratio. When the expected percent of time that two or more females are in estrus simultaneously is greater than about 5%, male-biased power is more probable than non male-biased power. Essentially, once a primate species exhibits even a small amount of estrous synchrony, then male-biased power is predicted as the most probable outcome.

**Figure 3:**
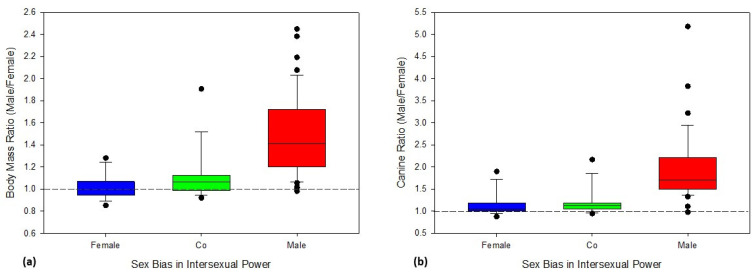
Sexual dimorphism in body mass and canine length does not necessarily constrain intersexual power relationships unless it is substantial. Species with non-male-biased power (female dominant (blue) and co-dominant (green)) tend to exhibit body masses near monomorphism (**a**) and relatively low degrees of male-biased canine dimorphism (**b**). Species with male-biased power (red) tend to exhibit much greater degrees of male-biased body mass and canine size dimorphism. No primate species exhibited a mean female body mass >18% larger than male body mass. With few exceptions, primate species in which males are ≥33% larger than females also exhibited male biased power structures. Dashed horizontal line: monomorphism, boxes: 25th/75th percentiles, lines: medians, whiskers: 10th/90th percentiles, dots: outliers.

To control for the possibility that species exhibiting extreme sexual dimorphism may have exerted a disproportionate influence on our results, we reran our analyses excluding species in which male body mass was more than approximately a third larger than females (Table 2; Figure 2e,f). Note that no primate exhibits extreme female-biased dimorphism and thus all excluded species exhibited male-biased dimorphism and nearly all exhibited male-biased power (Figure 3). The restricted analyses (Figure 2e,f) yield results that are similar to the more inclusive analyses (Figure 2c,d), and again suggest that very low expected estrous overlap and female-biased sex ratios were significantly associated with power that is not male-biased. The removal of the extremely dimorphic species accordingly did not have a substantial effect on the predicted probability of male-biased power (Figure 2).

## 4. Discussion

An individual’s physical attributes are an important factor shaping its social status, regardless of whether the unit of analysis is an encounter [83], a relationship [2,3,4,16,84], or the hierarchical structure of a social group [5,83,85,86]. However, physical attributes are not the only variable that influences social status. Power is an emergent phenomenon that can arise from many different asymmetries [2,3,5] and is embedded within a larger social, demographic, and economic context (see also [87]). This social context is particularly important for understanding intersexual power because male–female relationships often involve the exchange of services or commodities (e.g., [19,88,89]).

Our ASRs suggest that the likelihood of male-biased power in the LCAs of Primates, Strepsirrhini, and Haplorhini (Table 1) is sufficiently low (scaled likelihood = ~0.45–0.58) that one cannot meaningfully rule out other patterns of intersexual power. Based on these analyses (Figure 1), there is accordingly no reason to assume that male-biased power was the ancestral condition for primates, strepsirrhines, or haplorhines. By comparison, the LCAs of Anthropoidea, Platyrrhini, and Catarrhini are each reconstructed as having a high likelihood (scaled likelihood = ~0.84–0.95) of exhibiting male-biased power. However, these results for anthropoids and platyrrhines may not be attributable to high body mass dimorphism because the LCAs of both clades are reconstructed as being only slightly more dimorphic in body mass (BMRs = 1.15–1.16) than the primate, haplorhine, strepsirrhine, and lemuriform LCAs (BMRs = 1.10–1.14; Table 1). A shift to much higher canine dimorphism (i.e., male canine length more than 25% greater than female canine length) is reconstructed for the anthropoid LCA, suggesting that variables influencing canine dimorphism (e.g., male–male contest competition for mates: [90]) may have exerted a corresponding influence on anthropoid intersexual power relationships. Our analyses further suggest that considerably higher body mass dimorphism (i.e., males more than 25% greater than female body mass) subsequently evolved in the catarrhine LCA, which in turn may have further constrained intersexual power relationships among catarrhines in favor of male-biased power. Because many selective variables may influence male and female body mass independently [91,92], which factor(s) initially favored this increase in catarrhine body mass dimorphism is unclear. Irrespective of this uncertainty, if the LCA of extant Catarrhini was indeed highly dimorphic in both body mass and canine length, then the potential for males to exert power over females using force or the threat of force could be greater than in other primate clades. If so, this ancestral condition could help to explain why most catarrhine species are described by researchers as male dominant.

**Figure 4:**
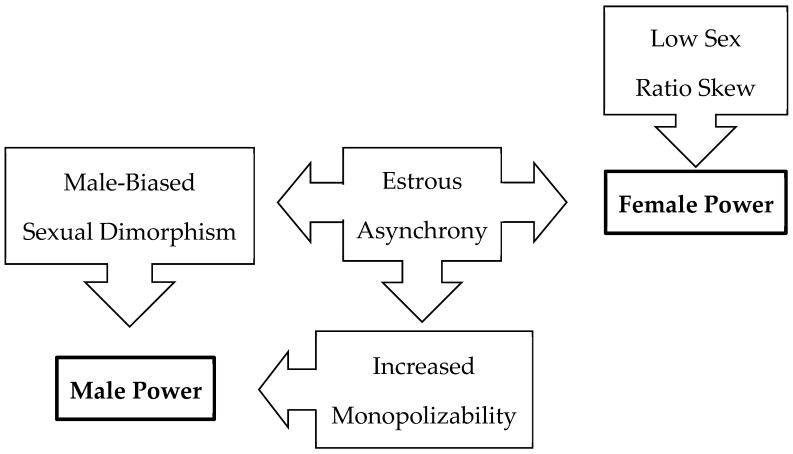
Male power is common because multiple traits lead to bias power structures towards males but few traits bias power structures towards females.

Moreover, male-biased power may be relatively common in primates because multiple traits lead to male-biased intersexual power, but few traits are associated with female-biased intersexual power or equality. Extremely low expected estrous overlap favors female power but may also lead to a greater potential for male monopolization and male contest competition [36]. These kinds of male contests for females tend to select for enhanced male armaments (e.g., longer canines) and greater sexual dimorphism in body mass [93], which are associated with reduced female intersexual power according to our analyses. Thus, female-biased power structures are primarily expected to evolve in species with low sexual dimorphism and little estrous synchrony among females (Figure 4). While *Erythrocebus*, with its extremely high dimorphism and non-male-biased power, might at first appear to contradict this general pattern, it has extremely short mating seasons and influxes of males into social groups when females are in estrus [94], suggesting that leverage indeed may explain the greater female power in this taxon.

Our ASRs further suggest that the LCA of Lemuriformes probably exhibited female-biased power (Figure 1). Whether this condition is apomorphic (derived) or plesiomorphic (primitive) for lemurs is uncertain because the pattern of intersexual power in the LCA of Strepsirrhini is unresolved. Nevertheless, the distribution of character states among extant species indicates that non-male-biased power structures have evolved numerous times among primates. When all of the variables analyzed here are considered together, it becomes apparent that the “lemur syndrome” of low dimorphism, fairly equal sex ratios, and power that is not biased towards males [9,30] occurs broadly among non-lemuriform primates in multiple clades (e.g., Hylobatidae, Callitrichinae, Aotinae, and Callicebinae). By comparison, a “catarrhine syndrome” of high dimorphism in body mass and canine size, highly skewed sex ratios, and male-biased power almost certainly represents a derived pattern for Primates (see also [95]).

The LCA of Primates is typically hypothesized to have been small, nocturnal, and solitary [96]. Thus, our reconstruction of the LCA of Primates as fairly monomorphic (Table 1) and with an unresolved pattern of intersexual power is not surprising. In fact, a solitary species may not have evolved the necessary condition of individual recognition for power *relationships*, as opposed to *interactions*, to occur ([2,4], see also [97]). Alternatively, the primate LCA may have lived in dispersed social networks with individual recognition among neighbors but fewer direct interactions than in more gregarious and day-active species [96]. If we are correct that it was not strongly dimorphic in body mass or canine size, then the primate LCA would have been free to exhibit a pattern of intersexual power more consistent with factors related to leverage effects (e.g., demographic variables influencing mating markets). Based on these considerations, the implicit but pervasive assumption in the primate literature that male-biased dimorphism and male-biased power structures are ancestral and normative for primates [5] is not supported by our analysis.

Sexual dimorphism can evolve due to natural selection or sexual selection [91,98,99]. When factors favor the evolution of larger male body size (e.g., male–male contest competition for mates), the downstream effect is a constraint on the opportunities for female-biased power structures to evolve. When males are not substantially larger than females, female leverage can arise due to market effects [2,21] or asymmetry between the sexes in resource value [20,100,101]. In primates, more variables appear to favor male-biased power than female-biased power (Figure 4). Importantly, sex-biased intersexual power structures per se do not appear to select for increased dimorphism because females in primate species with female-biased power do not exhibit substantially larger body or canine sizes than males (Figure 3). Similarly, studies of sexual selection have also shown that females in mammal species with “reversed sex roles” tend not to evolve armaments [102]. Additional research is needed to explore why males are more likely to evolve larger body size and canines (i.e., armaments) in response to contest competition for mates than females, including possible constraints of androgens and other sex hormones as mediators of both intersexual differences in anatomy and social behavior.

Our results are consistent with a growing body of literature indicating that power in animals is more than just dominance (*sensu* [2]). The social environment influences dyadic relationships. Power can arise from multiple different types of asymmetries in social relationships, such as dependency, inalienable resources, and knowledge, and hence is more complex than what might be expected based on inter-individual differences in fighting ability alone [2,3,84,85]. Indeed, we found that sex ratio—an *extrinsic* factor—was a strong predictor of intersexual power. Similarly, a study of Verreaux’s sifaka (*Propithecus verreauxi*) found that female leverage over males fluctuates with sex ratio [19]. Intersexual power in vervet monkeys (*Chlorocebus pygerythrus*) is dynamic and varies with sex ratio [103,104]. In spotted hyenas (*Crocuta crocuta*), patterns of sex-biased power partly emerge from sex differences in coalitionary support [15], that in turn derive from sex-biased dispersal [16]. Future research should explore the explanatory value of these and other social factors in primate power structures. Moreover, spotted hyenas exhibit low dimorphism, low estrous synchrony, and a female-biased power structure [105,106], suggesting that our model based on primates (Figure 4) may have broader applicability.

The relationship between dominance and leverage is currently poorly understood [2]. Our comparative analysis of intersexual relationships suggests that female leverage is constrained by male dominance. Although our reduced dataset, with the most extreme dimorphic species removed, resulted in similar predicted probabilities (Figure 2), Figure 3 suggests that patterns of power other than male-biased power structures are difficult to evolve when males are substantially larger than females. When males and females are similar in size, any pattern of intersexual power is possible. Further research is needed to determine the interaction between these two types of power.

Finally, our literature review revealed that few authors publish the basis for labeling a species with a particular sex-biased power structure, especially when the species is categorized as “male dominant”. Until more detailed information is available on the base, means, amount, and scope of power (i.e., power characteristics), our findings support the use of more general terms, such as power, rather dominance [2] and male-biased power structure rather than male dominance (cf. [5]). Future investigations into the evolutionary patterns of intersexual power will benefit from explicit and consistent methods and terminology.

## 5. Conclusions

Intersexual inequality is common in primate societies. Male-biased power is often assumed to be near universal, associated with male-biased sexual dimorphism (e.g., [107]), and despite researchers lamenting that the “unfortunate, misinterpreted stereotype” of male dominance ignores female leverage [108] (p. 27), has rarely been tested. Our comparative analysis confirms that male-biased power is a common feature of most primate societies, but it also highlights that societies without male-biased power are not limited to any particular clade or island. In contrast to arguments for the evolution of other patterns power that are taxon-specific (e.g., “lemur syndrome”: e.g., [30]; “self-domestication”: [109]), we provide an explanation for evolutionary patterns of intersexual power that is applicable to the entire order Primates and has potential explanatory power for other mammalian orders as well. By reconstructing ancestral states, we demonstrate that male-biased power cannot be assumed for the LCA of Primates and that a suite of characteristics common in catarrhines—high sexual dimorphism, highly skewed sex ratios, and male-biased power—is likely derived for Primates. Moreover, sex-biased power is often assumed to be a consequence of sexual dimorphism [9,45,107], but our study demonstrates that primate species with female-biased power structures do not exhibit large degrees of female-biased sexual dimorphism in either body size or canine length. By examining the evolutionary drivers of all patterns of intersexual power, not just the evolution of female-biased power, our research challenges common assumptions about the evolutionary inevitability of “male dominance”.

## Figures and Tables

**Table 1:** Ancestral state reconstruction presented as maximum likelihood estimates (MLE) (confidence intervals) of power categories, body mass dimorphism, canine dimorphism, and sex ratio based on extant data only. Predicted probability of male power based on predictive equations generated by logistic regression analyses (see Table 2).

	Power Category *	Body Mass Ratio (BMR)		Canine Ratio (CR)		Sex Ratio (SR)	
Node (LCA)	Scaled Likelihood of Male Power	Predicted Probability of Male Power	MLE of Reconstructed Body Mass Ratio	Predicted Probability of Male Power	MLE of Reconstructed Canine Ratio	Predicted Probability of Male Power	MLE of Reconstructed Sex Ratio
Primates	0.535	0.58 (0.55–0.60)	1.14 (1.00–1.30)	0.73 (0.67–0.78)	1.18 (0.92–1.51)	0.33 (0.44–0.23)	0.67 (0.48–0.94)
Strepsirrhini	0.447	0.58 (0.55–0.60)	1.14 (1.00–1.30)	0.71 (0.66–0.76)	1.09 (0.89–1.34)	0.31 (0.42–0.22)	0.70 (0.50–0.98)
Haplorhini ^#^	0.579	0.58 (0.55–0.60)	1.14 (1.00–1.30)				
Lemuriformes	0.018	0.57 (0.54–0.59)	1.10 (0.95–1.27)	0.71 (0.66–0.75)	1.05 (0.87–1.28)	0.28 (0.37–0.20)	0.79 (0.58–1.09)
Lorisiformes	0.569	0.57 (0.55–0.60)	1.14 (0.98–1.32)	0.70 (0.66–0.75)	1.05 (0.88–1.25)	0.32 (0.45–0.21)	0.69 (0.46–1.04)
Anthropoidea	0.844	0.58 (0.56–0.60)	1.16 (1.02–1.32)	0.75 (0.70–0.79)	1.27 (1.03–1.57)	0.36 (0.46–0.28)	0.60 (0.45–0.79)
Platyrrhini	0.876	0.58 (0.55–0.60)	1.15 (1.01–1.31)	0.74 (0.70–0.78)	1.23 (1.03–1.45)	0.35 (0.43–0.27)	0.63 (0.49–0.81)
Catarrhini	0.950	0.59 (0.57–0.62)	1.27 (1.09–1.48)	0.78 (0.73–0.81)	1.44 (1.18–1.77)	0.40 (0.49–0.31)	0.54 (0.41–0.70)
Cercopithecoidea	0.998	0.61 (0.58–0.63)	1.38 (1.18–1.61)	0.81 (0.77–0.84)	1.68 (1.38–2.06)	0.44 (0.53–0.35)	0.48 (0.37–0.61)
Hominoidea	0.927	0.61 (0.58–0.63)	1.36 (1.15–1.61)	0.77 (0.73–0.81)	1.40 (1.15–1.70)	0.39 (0.50–0.30)	0.54 (0.40–0.73)

* No reconstructed ratio is presented because we assessed power as a discrete variable. ^#^ Tarsiers are not included in the CR or SR datasets, and accordingly there is no haplorhine node in CR and SR ASRs.

**Table 2:** Results from logistic regression analyses predicting the probability of male-biased power.

Dataset	Independent Variable	Estimate	SE	*t*	Bootstrapped *p*-Value	Bootstrapped Mean of the Intercept (*b*_0_)	Bootstrapped Mean of Independent Variable (*b*_1_)
All primates	Body mass dimorphism ^a^	1.573	0.436	3.612	***	0.2134	1.6228
	Canine length dimorphism ^a^	2.678	0.636	4.214	***	0.8117	2.7104
	Expected estrous overlap ^b^	0.773	0.339	2.277	*	−0.5840	0.9596
	Sex ratio ^a^	−3.210	0.865	3.711	***	−1.2963	−3.2791
Excluding extremely	Expected estrous overlap ^b^	1.221	0.499	2.445	**	−0.6490	1.2333
dimorphic taxa ^c^	Sex ratio ^a^	−2.946	0.954	3.087	***	−1.2069	−3.2538

*** *p* < 0.001, ** *p* < 0.01, * *p* < 0.05, ^a^ ratio of male to female; ^b^ following [37]; ^c^ dataset reduced by excluding taxa with males more than 36% larger than females.

## Data Availability

The data presented in this study are available in Supplementary Materials here.

## Supplementary Materials

The following supporting information can be downloaded at: https://www.mdpi.com/article/10.3390/ani13233695/s1, Figure S1: Phylogenetic tree; Table S1: Extant primate data [9,27,36,43,45,48,49,50,51,52,107,110,111,112,113,114,115,116,117,118,119,120,121,122,123,124,125,126,127,128,129,130,131,132,133,134,135,136,137,138,139,140,141,142,143,144,145,146,147,148,149,150,151,152,153,154,155,156,157,158,159,160,161,162,163,164,165,166]; Table S2: Dermoptera and Scandentia body mass ratio data [62,63,64]; Table S3: Fossil divergence dates and canine ratios [75,76,79,167,168,169,170,171,172,173,174,175,176,177,178,179].
