# Supplementary material for: Evolutionary Patterns of Intersexual Power
Source: Animals (Basel). 2023 Nov 29;13(23):3695. doi: 10.3390/ani13233695 (PMC10705298; doi:10.3390/ani13233695)
Supplement: Supplementary file 1 [file animals-13-03695-s001.zip › animals-2666281-supplementary.pdf]

Figure S1. Phylogenetic tree

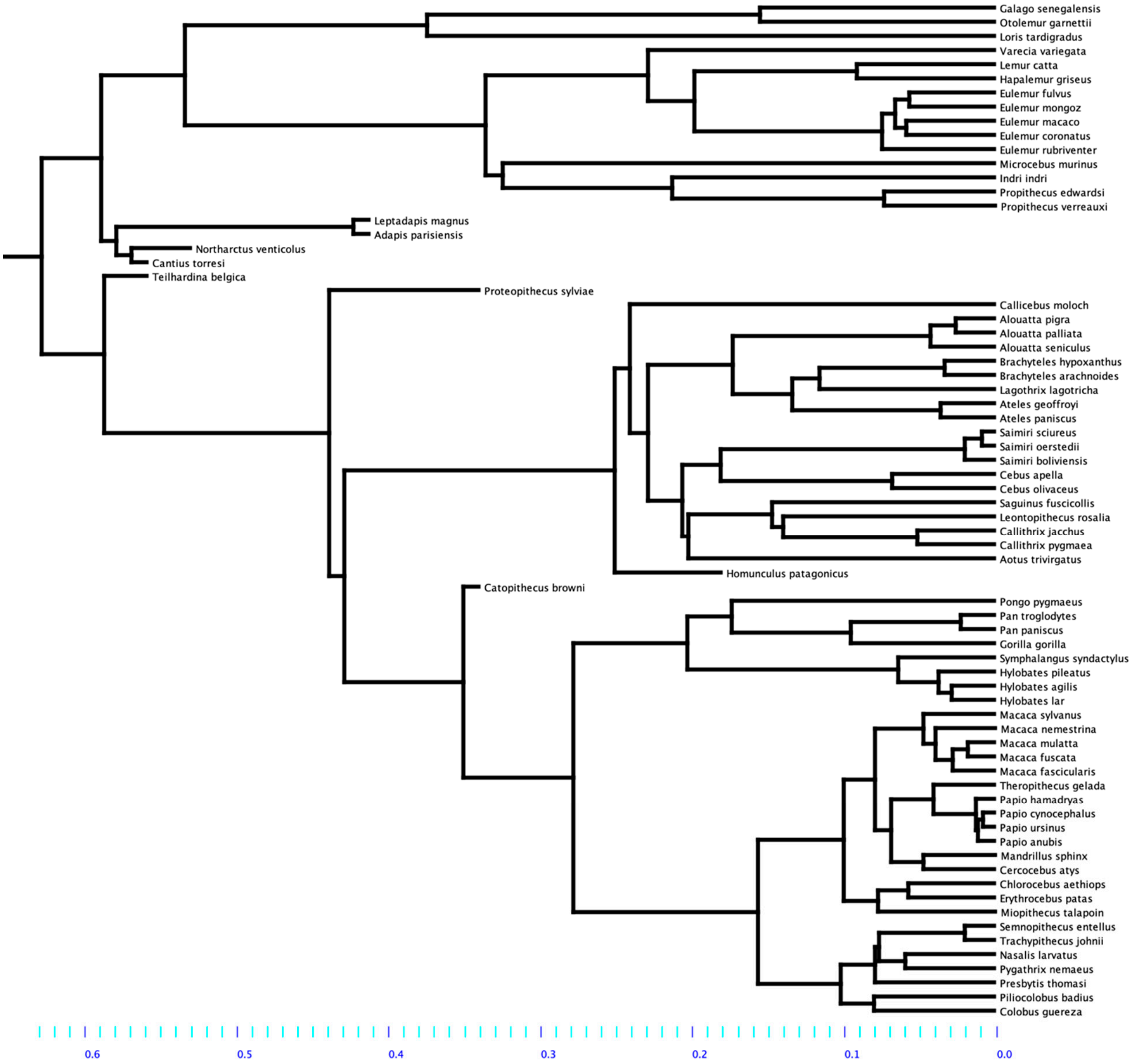

Phylogenetic tree (above) generated using the Nexus file (below) employed in ancestral state reconstructions of canine ratio. In the scale at bottom, 0.1 = 10 million years before present. See main text for details regarding estimated clade divergence times.

#NEXUS
[written Tue May 03 11:42:50 EDT 2016 by Mesquite version 2.74 (build 550) at Ashley-Gosselin-Ildaris-MacBook-Pro-2.local/192.168.0.63]

BEGIN TAXA;

```
	TITLE Taxa;
	DIMENSIONS NTAX=72;
	TAXLABELS
		Galago_senegalensis Otolemur_garnettii Loris_tardigradus Varecia_variegata
Lemur_catta Hapalemur_griseus Eulemur_fulvus Eulemur_mongoz Eulemur_macaco
Eulemur_coronatus Eulemur_rubriventer Microcebus_murinus Indri_indri
Propithecus_edwardsi Propithecus_verreauxi Callicebus_moloch Alouatta_pigra
Alouatta_palliata Alouatta_seniculus Brachyteles_hypoxanthus Brachyteles_arachnoides
Lagothrix_lagotricha Ateles_geoffroyi Ateles_paniscus Saimiri_sciureus Saimiri_oerstedii
Saimiri_boliviensis Cebus_apella Cebus_olivaceus Saguinus_fuscicollis Leontopithecus_rosalia
Callithrix_jacchus Callithrix_pygmaea Aotus_trivirgatus Pongo_pygmaeus Pan_troglodytes
Pan_paniscus Gorilla_gorilla Symphalangus_syndactylus Hylobates_pileatus Hylobates_agilis
Hylobates_lar Macaca_sylvanus Macaca_nemestrina Macaca_mulatta Macaca_fuscata
Macaca_fascicularis Theropithecus_gelada Papio_hamadryas Papio_cynocephalus
Papio_ursinus Papio_anubis Mandrillus_sphinx Cercocebus_atys Chlorocebus_aethiops
Erythrocebus_patas Miopithecus_talapoin Semnopithecus_entellus Trachypithecus_johnii
Nasalis_larvatus Pygathrix_nemaeus Presbytis_thomasi Piliocolobus_badius Colobus_guereza
Catopithecus_browni Proteopithecus_sylviae Homunculus_patagonicus Leptadapis_magnus
Adapis_parisiensis Cantius_torresi Teilhardina_belgica Northarctus_venticolus
	;

END;


BEGIN TREES;
	TRANSLATE
		1 Galago_senegalensis,
		2 Otolemur_garnettii,
		3 Loris_tardigradus,
		4 Varecia_variegata,
		5 Lemur_catta,
		6 Hapalemur_griseus,
		7 Eulemur_fulvus,
		8 Eulemur_mongoz,
		9 Eulemur_macaco,
		10 Eulemur_coronatus,
		11 Eulemur_rubriventer,
		12 Microcebus_murinus,
		13 Indri_indri,
		14 Propithecus_edwardsi,
		15 Propithecus_verreauxi,
		16 Callicebus_moloch,
		17 Alouatta_pigra,
		18 Alouatta_palliata,
```

19 Alouatta_seniculus,
20 Brachyteles_hypoxanthus,
21 Brachyteles_arachnoides,
22 Lagothrix_lagotricha,
23 Ateles_geoffroyi,
24 Ateles_paniscus,
25 Saimiri_sciureus,
26 Saimiri_oerstedii,
27 Saimiri_boliviensis,
28 Cebus_apella,
29 Cebus_olivaceus,
30 Saguinus_fuscicollis,
31 Leontopithecus_rosalia,
32 Callithrix_jacchus,
33 Callithrix_pygmaea,
34 Aotus_trivirgatus,
35 Pongo_pygmaeus,
36 Pan_troglodytes,
37 Pan_paniscus,
38 Gorilla_gorilla,
39 Symphalangus_syndactylus,
40 Hylobates_pileatus,
41 Hylobates_agilis,
42 Hylobates_lar,
43 Macaca_sylvanus,
44 Macaca_nemestrina,
45 Macaca_mulatta,
46 Macaca_fuscata,
47 Macaca_fascicularis,
48 Theropithecus_gelada,
49 Papio_hamadryas,
50 Papio_cynocephalus,
51 Papio_ursinus,
52 Papio_anubis,
53 Mandrillus_sphinx,
54 Cercocebus_atys,
55 Chlorocebus_aethiops,
56 Erythrocebus_patas,
57 Miopithecus_talapoin,
58 Semnopithecus_entellus,
59 Trachypithecus_johnii,
60 Nasalis_larvatus,
61 Pygathrix_nemaeus,
62 Presbytis_thomasi,

63 Piliocolobus_badius,
64 Colobus_guereza,
65 Catopithecus_browni,
66 Proteopithecus_sylviae,
67 Homunculus_patagonicus,
68 Leptadapis_magnus,
69 Adapis_parisiensis,
70 Cantius_torresi,
71 Teilhardina_belgica,
72 Northarctus_venticolus;

TREE 'tree 1++++++++' =
(((((1:0.15424,2:0.154239):0.219241,3:0.37348000000000003):0.159386,((4:0.227883,((5:0.090545,6:0.090545):0.10682,(((7:0.056351,8:0.05635):0.008869,(9:0.057816,10:0.057816):0.007403):0.008732,11:0.073952):0.123414):0.030518):0.106967,(12:0.324026,(13:0.21206,(14:0.07302900000000001,15:0.07303):0.139031):0.111967):0.010823):0.198018):0.055134,((68:0.01,69:0.01):0.156001,(72:0.038001,70:0.01):0.01):0.01):0.03966,(71:0.028023,(66:0.097996,(((16:0.240312,((((17:0.025391,18:0.025391):0.016791,19:0.042183):0.130482,(((20:0.032681,21:0.032681):0.082851,22:0.115532):0.017714,(23:0.035505,24:0.035506):0.097741):0.039418):0.055489,((((25:0.008274,26:0.008274):0.011553,27:0.019826):0.160509,(28:0.067434,29:0.067434):0.1129):0.025303,((30:0.146636,(31:0.13928800000000002,(32:0.050905,33:0.050904):0.08838299999999999):0.007349):0.054989,34:0.201626):0.004012):0.022515):0.012159):0.01,67:0.070312):0.177684,(65:0.01,(((35:0.173188,((36:0.021921,37:0.021921):0.072787,38:0.094708):0.078479):0.029005,(39:0.063339,(40:0.037134,(41:0.028469999999999995,42:0.028469):0.008665):0.026205):0.138853):0.07507,((((43:0.046727,(44:0.038615,((45:0.017611,46:0.017611):0.010008,47:0.027619):0.010995):0.008113):0.032155,((48:0.0399,(49:0.012198,((50:0.007397,51:0.007397):0.003762,52:0.011159):0.001039):0.027700999999999996):0.028336,(53:0.046519000000000005,54:0.046518000000000004):0.021718):0.010646):0.020377,((55:0.056933000000000004,56:0.056933):0.019462,57:0.07639599999999999):0.022864):0.056418,((((58:0.019382,59:0.019382):0.056671000000000006,(60:0.058943999999999996,61:0.058943999999999996):0.017109):0.002503,62:0.078556):0.023016,(63:0.07952600000000001,64:0.079526):0.022046):0.054105):0.121586):0.072738):0.077995):0.01):0.148027):0.041639):0.045854;

end;

Table S1. Extant primate data [1]

Species	Dominance	Body Mass Ratio [48,49]	Canine Ratio [50-52]	Expected Estrus Overlap [36]	Sex Ratio [36]
Alouatta palliata	male[110]	1.3364	1.51	0.7	0.25
Alouatta pigra	male[2]	1.7729	1.37		
Alouatta seniculus	male[111]	1.2841	1.5	0.1	0.60
Aotus trivirgatus	co-dominance[112]	1.1046	1.08	0	1
Ateles belzebuth	male[111]	1.0561			

Ateles geoffroyi	male[111]	1.0672	1.52		
Ateles paniscus	male[111]	1.0794	1.57	9.6	0.31
Brachyteles arachnoides	male[113]	1.2000	1.33	5.7	0.65
Brachyteles hypoxanthus	co-dominance[111]	1.1309	1.12		
Cacajao rubicundus	male[114]	1.1979			
Callicebus moloch	co-dominance[115]	1.0669	1.08	0	1
Callicebus personatus	co-dominance[116]	0.9203			
Callithrix jacchus	co-dominance[117]	0.9784	0.95	6.3	0.93
Cebuella pygmaea	female[118]	0.9016	1.03	0	1
Cebus albifrons	male[119]	1.3886			
Cebus apella	male[120]	1.4484	1.41	1.2	0.87
Cebus capucinus	male[121]	1.4488		1.5	1.38
Cebus olivaceus	male[120]	1.3056	1.57	9.3	0.17
Cercocebus atys	male[122]	1.7742	2.46		
Chlorocebus aethiops	male[107]	1.4295	1.51	92	0.70
Cercopithecus campbelli	male[123]	1.6700			
Colobus guereza	male[124]	1.4674	1.46	0.6	0.33
Erythrocebus patas	co-dominance[45]	1.9077	2.17	20	0.23
Eulemur coronatus	female[125]	1.0432	1.11		
Eulemur fulvus	co-dominance[43]	0.9689	1.18	8.4	1.36
Eulemur macaco	female[126]	0.9442	1.02	5.1	0.91
Eulemur mongoz	female[127]	1.0124	1.2	0	1
Eulemur rubiventer	female[128]	1.0206	1.05	0	1
Galago senegalensis	male[129]	1.1407	1.11		
Gorilla gorilla	male[130]	2.3832	1.73	0.2	0.41
Hapalemur griseus	female[131]	1.0930	1	0	1
Hylobates agilis	co-dominance[132]	1.0103	1.08		
Hylobates lar	co-dominance[132]	1.1049	1.16	0	1
Hylobates muelleri	co-dominance[132]	1.0673			
Hylobates pileautus	co-dominance[132]	1.0110	0.99		
Hylobates syndactylus	co-dominance[133]	1.1121	1.18		
Indri Indri	female[134]	0.8523	1	0	1
Lagothrix lagothricha	male[111]	1.1634	1.77	4.3	0.34
Lemur catta	female[135]	1.0000	1.19	1.5	1.13
Leontopithecus rosalia	co-dominance[136]	1.0368	1.18	2.9	1.33
Loris tardigradus	male[137]	0.9814	0.98	0	0.40
Macaca assamensis	male[3]	1.6377			
Macaca fascicularis	male[138]	1.4930	2.25	54	0.59
Macaca fuscata	male[139]	1.3699	1.94	97	0.33
Macaca mulatta	male[140]	1.2500	2.15	62	0.28
Macaca nemestrina	male[141]	1.7200	2.35	47	0.14

Macaca radiata	male[142]	1.7325		26	0.78
Macaca sylvanus	male[4]	1.4545	2.02	95	0.82
Macaca thibetana	male[143]	1.6000			
Mandrillus sphinx	male[144]	2.4496	5.18		
Microcebus murinus	female[27]	1.0575	0.99	0.2	0.57
Miopithecus talapoin	female[9,145]	1.2321	1.9	100	0.48
Nasalis larvatus	male[146]	2.0774	2.2		
Otolemur garnetti	female[147]	1.0817	1.08	0	1
Pan paniscus	co-dominance[148]	1.3554	1.38	14	1
Pan troglodytes	male[149]	1.2671	1.43	76	0.29
Papio anubis	male[150]	1.8872	2.22	31	0.41
Papio cynocephalus	male[151]	1.7724	2.83	13	0.62
Papio hamadryas	male[152]	1.7071	2.74	0.1	0.50
Papio papio	male[153]	1.9167			
Papio ursinus	male[154]	2.0135	3.83	17	0.47
Pongo pygmaeus	male[107]	2.1927	1.7	79	0.56
Presbytis thomasi	male[138]	1.0120	1.8		
Piliocolobus badius	male[155]	1.0183	1.88	14	0.50
Propithecus diadema	female[125]	0.9489			
Propithecus edwardsi	female[156]	0.9649	0.88	0	1.07
Propithecus tattersalli	female[125]	0.9443			
Propithecus verreauxi	female[157]	0.9438	1.06	0.3	1
Pygathrix nemaeus	male[158]	1.3033	2.02		
Saguinas fuscicollis	co-dominance[159]	0.9581	1.03	0	1.90
Saimiri boliviensis	female[160]	1.2813	1.61		
Saimiri oerstedii	male[161]	1.3191	1.68	30	0.63
Saimiri sciureus	male[162]	1.1767	1.41	45	0.30
Semnopithecus entellus	male[163]	1.3145	1.7	6.4	0.32
Tarsius tarsier	co-dominance[5]	1.1574			
Theropithecus gelada	male[164]	1.6239	3.22	1.4	0.25
Trachypithecus johnii	male[165]	1.0714	1.63		
Trachypithecus phayrei	male[6]	1.2492			
Varecia variegata	female[166]	0.9886	1.04	0	0.50

[1] Numbers in brackets refer ro references in the main body of the manuscript

[2] Sarie Van Belle Personal communication

[3] Sally Macdonald Personal communication

[4] Christopher Young Personal communication

[5] Sharon Gursky Personal communication

[6] Andreas Koenig and Carola Borries Personal communication

Table S2. Dermoptera and Scandentia body mass ratio data [62–64]

Species	Body Mass Ratio
Galeopterus variegatus	1
Cynocephalus volans	1
Ptilocercus lowii	0.92
Tuapaia longipes	1.01
Tupaia gracilis	0.96
Tuapaia minor	1.04
Tupaia montana	1.02
Tupaia tana	0.94

Table S3: Fossil divergence dates and canine ratios.

Taxon	Fossil Age (mya)	Fossil Age (epoch)	Reference	Computed Divergence (mya)	Assigned Branch Length	Canine Ratio	Reference
Leptadapis magnus	41.2	Lutetian-Bartonian boundary (Eocene)	[79,167,168]	42.4	1	1.19	[175]
Adapis parisiensis	41.2	Lutetian-Bartonian boundary (Eocene)	[79,167,168]	42.2	1	1.13	[175]
*Cantius torresi**	55.8	WA-0 (earliest Eocene)	[169,170]	56.8	1	1.37	[176]
Northarctus venticolus	53	BR-0 (early Eocene)	[170,171]	56.8	3.8	1.89	[177]
Catopithecus browni	34	Jebel Qatrani Formation (late Eocene - early Oligocene)	[172]	35	1	1.43	[178]
*Proteopithecus sylviae**	34	Jebel Qatrani Formation (late Eocene - early Oligocene)	[172]	43.8	9.8	1.28	[178]
Homonculus patagonicus	18	Santa Cruz Formation (Miocene)	[173]	25	7	1.28	[179]
Teilhardina belgica~	55.8	WA-0 (earliest Eocene)	[170,174]	58.6	2.8	1	[75,76]

*Canine Ratio based on mandibular canines

~Canine Ratio not reported; Omomyiforms reported as monomorphic in canine ratio
